# Safety, Acceptability, and Use of a Smartphone App, BlueIce, for Young People Who Self-Harm: Protocol for an Open Phase I Trial

**DOI:** 10.2196/resprot.6525

**Published:** 2016-11-16

**Authors:** Paul Stallard, Joanna Porter, Rebecca Grist

**Affiliations:** ^1^ Child and Adolescent Mental Health Group Department for Health University of Bath Bath United Kingdom; ^2^ Child and Family Mental Health Temple House Oxford Health National Health Service Foundation Trust Keynsham United Kingdom

**Keywords:** self-harm, smartphone app, BlueIce, adolescents, CBT, DBT

## Abstract

**Background:**

Up to 18% of adolescents will engage in an act of self-harm before young adulthood, with the majority of acts occurring in private. Mobile apps may offer a way of providing support for young people at times of distress to prevent self-harm.

**Objective:**

This is a proof-of-concept study designed to explore the safety, acceptability, feasibility, and usability of a smartphone app, BlueIce, with young people who are self-harming.

**Methods:**

In this phase I open trial we will evaluate BlueIce, a smartphone app developed and coproduced with young people with lived experience of self-harm. BlueIce includes a mood-monitoring diary, selection of mood-lifting techniques based on cognitive behavior therapy and dialectical behavior therapy, and direct access to emergency telephone numbers. We will recruit young people (n=50) attending specialist child and adolescent mental health services with a current or past history of self-harm to trial BlueIce as an adjunct to their usual care. Questionnaires and interviews will be completed at baseline, postfamiliarization (2 weeks), and at follow-up (12 weeks after baseline) to assess safety, app use, and acceptability. Interviews will be undertaken with clinicians to assess the feasibility of BlueIce within a clinical setting.

**Results:**

Recruitment occurred between May and November 2016. The recruitment target was 50, and by the beginning of November 54 young people had been referred.

**Conclusions:**

This study is the first to evaluate an app specifically developed with young people for young people (under the age of 18 years) who self-harm. It will determine whether BlueIce is acceptable, how often it is used, and whether it is safe and does not have any unintentional adverse effects. This information will determine whether a feasibility trial to test recruitment, randomization, retention, and appropriate outcome measures should be pursued.

## Introduction

### Overview

Self-harm is defined as intentional self-poisoning or self-injury, irrespective of type of motive or the extent of suicidal intent [1]. Self-harm is a risk factor for suicide. Although suicide rates in those under the age of 18 years are comparatively low, approximately half of those who commit suicide have been found to have a previous history of self-harm [2]. However, the majority of self-harm in adolescence is self-destructive and often occurs without suicidal intent (nonsuicidal self-injury).

While suicide rates in this age group are low, self-harm is unfortunately common with community studies from many countries consistently reporting a lifetime risk of 13% to 18% [3-7]. Of those who self-harm, half will report multiple self-harming events [6]. For example, in a UK community survey of young people aged 12 to 16 years from 8 schools, 15% reported acts of self-harm over the past 12 months with 55% reporting self-harm over 2 consecutive 6-month episodes [8].

In community surveys in developed countries, self-cutting is the most commonly reported method of self-harm whereas self-poisoning is more common in those who present at accident and emergency departments [5,6,9]. However, comparatively few episodes of self-harm result in hospital presentations with most being undertaken in private and remaining hidden [10].

Although suicide is comparatively uncommon in adolescents, it is the second or third leading cause of death within this age group [10]. Self-harm is associated with an increased risk of mortality and suicide. A 10-year follow-up study in the United Kingdom of young people who presented at hospital following an episode of self-harm found that of those who subsequently died, half had committed suicide [11].

In terms of risk factors, self-harm is associated with a range of sociodemographic and educational factors (eg, gender, lower socioeconomic status, sexual orientation), life events (eg, trauma, abuse, family breakdown), and psychological factors (eg, depression, drug and alcohol misuse, impulsivity) [10].

### Interventions for Self-Harm

A UK review by the National Institute of Health and Care Excellence (NICE) recommends that all children under 16 years of age attending hospital following self-harm should be admitted to a pediatric ward and assessed by an experienced mental health practitioner [1]. In terms of interventions, NICE recommends a 3- to 12-session psychological intervention with the aim of reducing self-harm. NICE recommends that it is tailored to individual need and could include cognitive behavioral, psychodynamic, or problem-solving elements [1]. However, comparatively few trials of interventions for children and adolescents who self-harm have been reported. A recent UK Cochrane review concluded that “there is not much evidence on which to draw conclusions on the effects of interventions for self-harm in this population“ [12]. The review found little support for group-based therapy but suggested that therapeutic assessment, mentalization, and dialectical behavior and cognitive behavior therapy warrant further evaluation. Finally, it recommended that any new therapeutic interventions should be developed in collaboration with patients to ensure patients’ needs are met.

### Telemedicine

Telemedicine is the use of information and communication technologies to increase access to care and improve health outcomes [13]. Adolescents are familiar with and frequent users of technology. A survey by the Office of Communications, the regulatory body supervising the communications industry in the United Kingdom, found that nearly all young people aged 12 to 15 years (98%) have Internet access [14]. Approximately 90% of 15-year-olds have a smartphone with ownership increasing with age. Smartphones therefore offer an accessible way of delivering and supporting mental health interventions for this age group.

One particular area that has seen a phenomenal expansion in recent years is the development of smartphone apps. Increasingly these have been developed to help with a range of mental health problems [15]. However their development has significantly outpaced research, and the evidence for their efficacy is largely unknown [15]. In terms of self-harm, no smartphone apps have been specifically developed for young people (under the age of 18) who self-harm.

### BlueIce Development

The development of BlueIce has followed the Medical Research Council framework for the development and evaluation of complex interventions [16]. The original idea was discussed with a group of young people who had a lived experience of self-harm. The young people thought that a smartphone app could improve self-management and provide a helpful way to manage distress and prevent self-harm at times of crisis.

The content of the app was informed by theoretical approaches that appeared promising in treating self-harm—in particular, cognitive behavior therapy (CBT) and dialectical behavior therapy (DBT). The structured nature of these therapies facilitated the incorporation of core therapeutic techniques into a mobile app. BlueIce therefore includes ideas from CBT (behavioral activation, thought challenging, and mood lifting activities) and DBT (mindfulness and distress tolerance).

Further meetings with young people focused on app content, design, and presentation. A beta version of BlueIce was produced which was reviewed by the young people and a group of child mental health professionals. Further recommendations for content and design were suggested and these were incorporated into the second beta version. This was reviewed again with the young people who positively endorsed the app.

BlueIce was developed for Android operating systems and was coded natively. Although it does not connect or interact with existing health care systems, data stored on BlueIce can be reviewed with the young person’s mental health clinician during routine clinical appointments.

### Aims of the Study

The aim of this study is to undertake a phase I trial to explore the safety, acceptability, feasibility, and usability of BlueIce with young people aged 12 to 18 years who are self-harming.

## Methods

### Study Design

This is a phase I open trial where eligible young people will be invited by their mental health clinician to use BlueIce. The study was funded by the Health Foundation (2143 Oxford Health National Health Service [NHS] Foundation Trust), and the protocol was approved by the NHS South West—Exeter Research Ethics Committee (reference 16/SW/0018).

### Setting and Participants

The study will be undertaken in specialist child and adolescent mental health services (CAMHS) provided by Oxford Health NHS Foundation Trust. The Trust serves a wide geographical area that includes Bath and North East Somerset, Buckinghamshire, Oxfordshire, Swindon, and Wiltshire.

Eligible participants will be aged 12 to 18 years with a history of repeated self-harm. Participants may be currently self-harming (within the past 4 weeks) or have a history of self-harm and feel that they will harm themselves again. BlueIce is designed to be used alongside specialist CAMHS so young people must be in receipt of an ongoing face-to-face intervention.

Young people will be excluded if they have active suicidal ideation and are seriously contemplating or planning a suicide attempt. Given that we do not know whether BlueIce will have any unintentional adverse consequences, it would not be safe to test it with a high-risk group who are actively suicidal. Second, young people will be excluded if they are diagnosed with psychosis or have a significant learning disability which might impede their ability to use the app. Third, we will exclude young people who have been subject to abuse within the last 6 months or are the subject of a safeguarding investigation. Finally, BlueIce is only available in English and we will therefore exclude those who are unable to understand English.

### Recruitment and Consent

Project information will be provided to all clinical teams and staff across Oxford Health. This will be followed by meetings with clinical teams and interested clinical staff to demonstrate BlueIce. Clinicians will be provided with project information sheets which they will be asked to discuss with eligible young people whom they think will benefit from BlueIce. The clinician will pass details of interested young people to the research team. The researcher will contact the young person to discuss the project and obtain written consent. For those under the age of 16, parental consent will also be required.

### Intervention

BlueIce is an Android smartphone app that has been coproduced with young people who have self-harmed. It contains a personalized toolbox of strategies that are available to the young person 24/7. It is designed to be used as an adjunct to therapy and includes a mood diary, a section of personalized mood-lifting techniques, and emergency contact numbers.

#### Mood Diary

Young people are able to monitor their mood each day. For each mood rating, they have the option of adding a note to record any particular reason why they might be feeling as they do. Their rating and notes are saved in a calendar which the young person and therapist can review to look for changes and patterns over time.

#### Mood Lifting

Young people who rate their mood as low will automatically be routed to the mood-lifting section of BlueIce. They can access this at any time directly from the main menu. This section contains a menu of mood-lifting activities personalized according to the interests of the young person. The activities are designed to counter the common reasons why young people self-harm (to punish themselves, emotional relief, feeling hopeless) and draws on common methods used in CBT and DBT. The mood-lifting section includes 8 activities:

- Photographs, inspirational quotes, and pictures that are associated with happy memories can be uploaded and saved. These can be reviewed when low to help young people remember the positive things in their life.
- A music player is included where young people can upload and store music they enjoy and which has a positive effect on how they feel. This playlist can be readily accessed as a way of improving their mood.
- Young people can create a personalized list of physical activities they enjoy like going for a run or riding a bike or playing with siblings for review when they are low.
- Young people can create a list of activities—like making a cake, watching an episode of a favorite TV series, reading a book, playing with a pet—that can be reviewed when they are feeling down.
- Audio-recorded instructions for a 10-minute mindfulness session, calming visualization, and a quick controlled breathing exercise (4-7-8 breathing) are included. These can be used to help young people manage any unpleasant emotions or distressing thoughts.
- Young people can record any troubling thoughts that are racing through their heads into the thought diary. These can be directly typed into BlueIce where they are saved and can be reviewed with the clinician at a later date. This allows identification of any particular themes that could be addressed during face to face work with their clinician.
- This section draws on ideas from DBT and helps young people tolerate their distress. This includes instructions for an ice dive, a sensory toolbox, and a pros and cons balance sheet for self-harming.
- The final section contains the phone numbers of 3 to 5 people whom the young people could contact if they were feeling low and in danger of self-harming. These would be people who make them feel happy and those they could talk with about how they are feeling. This section prompts the young person to reach out to others.

#### Emergency Contacts

The final section contains phone numbers young people can call which provide direct access to emergency support both nationally and locally.

#### Study Procedure

**Figure 1:**
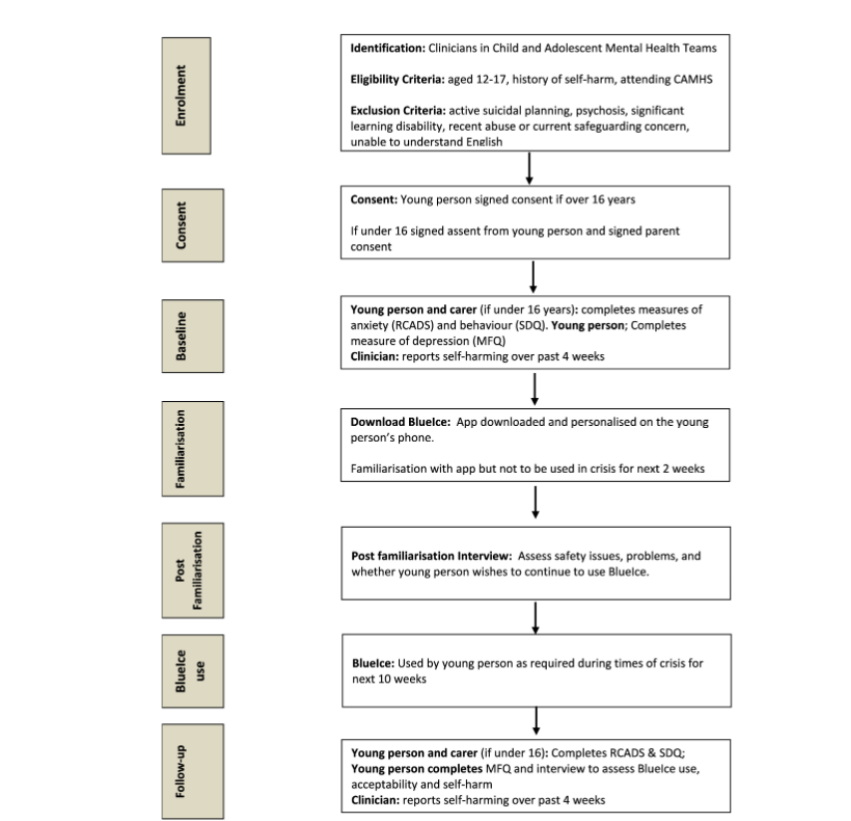
BlueIce study procedure. CAMHS: Child and Adolescent Mental Health Services; RCADS: Revised Child Anxiety Scales; SDQ: Strengths and Difficulties Questionnaire; MFQ: Mood and Feelings Questionnaire.

The study procedures are summarized in Figure 1. Clinicians will be invited to identify and discuss BlueIce with young people they were working with who meet inclusion criteria. If the young person is interested the clinician will contact the research team and inform them whether the young person has self-harmed in the last 4 weeks.

The research team will arrange to meet with the young people and, if under the age of 16 years, their carers. The study will be discussed, informed consent obtained, and baseline assessments completed. BlueIce will then be downloaded on to the young person’s phone and the sections in the mood lifter personalized.

In order to ensure safety, young people will be instructed to familiarize themselves with BlueIce but not to use it at times of crisis for the next 2 weeks. BlueIce will then be reviewed with the researcher after familiarization to discuss how they found the app and whether they encountered any problems. The young person will then choose to either continue using BlueIce for the next 10 weeks or to stop and have it removed from the phone. If they chose to use BlueIce, they can use it as often as they wish. Follow-up assessments will be completed with young people and the use and acceptability of BlueIce determined.

### Outcome Measures

#### Mood

Young people and their carers (if under 16 years of age) will complete the Revised Child Anxiety Scales (RCADS) [17-19] and Strengths and Difficulties Questionnaire (SDQ) [20]. These provide an assessment of anxiety, depression, and behavioral problems. Young people will also complete the Mood and Feelings Questionnaire (MFQ) [21], a standardized measure to assess depression. All questionnaires will be completed at baseline and at follow-up after using BlueIce.

#### Safety

Semistructured interviews will be undertaken with young people postfamiliarization and at follow-up. These will focus on safety and in particular whether BlueIce worked as intended (ie, did not crash or freeze) and had no unintentional adverse effects. For example, constantly recording mood as low might increase thoughts of hopelessness and self-harm. Young people will also be asked to rate the extent to which BlueIce might make them self-harm and whether it might help.

#### Acceptability

Although BlueIce was developed with young people who had a lived experience of self-harm, we will assess whether the BlueIce layout and content are understandable and acceptable to those currently in crisis. This will be assessed during postfamiliarization and follow-up interviews and through ratings assessing ease of use, helpfulness, and whether to recommend BlueIce to a friend.

#### Usability

We will assess whether BlueIce can be easily downloaded and personalized on different Android phones. We will also check which parts of the app young people use and which they find most helpful.

#### Self-Harm

Information will be obtained from referring clinicians about whether the young person had been self-harming in the 4 weeks before using BlueIce and in the 4 weeks before the follow-up assessment. In addition, young people will be asked during the follow-up interview whether BlueIce had helped to prevent an episode of self-harm and if so, how many.

#### Feasibility

This will be assessed through the number of referrals per team and professional group and through interviews with referring clinicians. We will also undertake qualitative interviews with clinicians to determine usability, usefulness, and fit with clinical practice.

### Sample Size

This is an initial feasibility study and as such no formal power calculation was undertaken. For this phase I safety trial we plan to recruit 50 young people, a sample that will be sufficient to reach saturation through analysis of data collected in semistructured interviews [22].

### Statistical Analysis

We will describe the cohort in terms of age, gender, and previous self-harm and report recruitment, completion, and drop-out rates. Safety will be assessed by the percentage of participants who report problems, a worsening of mood, or an increase in self-harm. Mean and standard deviations of ratings (potential harm and help) will be reported.

Acceptability will be determined by satisfaction ratings and through analysis of semistructured interviews. Interviews will be audiorecorded and transcribed. They will then be analyzed using a predefined framework derived from the interview schedule and adapted and revised on the basis of participant responses [23]. The interviews will also assess usability and how often young people used the app and which features.

Feasibility will be assessed by examining referral flows by teams and professional groups. We will also interview referring clinicians to obtain their views about usability, usefulness, and fit with clinical practice.

Outcome data from standardized questionnaires and reported changes in self-harm will be treated as preliminary and will not be subject to extensive analysis. We will conduct an exploratory analysis using descriptive statistics, where appropriate, to report pre- and post-use changes.

## Results

We have completed recruitment and are now undertaking post-use assessments.

## Discussion

This study seeks to explore the safety, acceptability, feasibility, and use of a smartphone app for young people attending specialist CAMHS who are self-harming. Our study addresses an important problem and through the use of technology aims to empower young people to manage their unpleasant emotions and thoughts of self-harm at times of crisis. A particular strength of our intervention is the coproduction with young people who have a lived experience of self-harm. Young people have shaped the content, design, and appearance of BlueIce in order to ensure that it is acceptable and attractive to our intended users. A further strength of our design is the focus on evaluation and in determining safety. It is important that smartphone apps used as adjuncts to mental health interventions are subject to evaluation [24,25]. In particular, when dealing with sensitive issues such as self-harm, it is important to demonstrate that apps are safe and do not unintentionally cause any adverse events.

To our knowledge, this is the first smartphone app designed with and produced for young people under the age of 18 years who self-harm. Our study design does however have limitations—in particular our reliance on clinical staff to identify and recruit young people. Clinicians have been found to have negative views about eMental health as highlighted in studies looking at clinician attitudes to computerized interventions [26,27]. We are unsure about clinicians’ views about smartphone apps as adjuncts to face-to-face care but are aware that uptake may be limited. We will attempt to minimize this risk through an active process of clinician engagement involving information-giving, demonstrations, and on-going email updates and informal meetings.

A second limitation is our reliance on retrospective self-report to assess the frequency of self-harm. Self-report may underestimate the number of events recalled, particularly for those young people who are engaging in regular and frequent harming. This limitation is acknowledged; although at this stage we are primarily focused on the app functionality and in assessing use, acceptability, and safety. Prospective reporting of self-harm (eg, through diaries) and objective measures of self-harm (eg, accident and emergency attendances) will be considered in a subsequent phase II trial.

## Abbreviations

CAMHS: Child and Adolescent Mental Health Services
CBT: cognitive behavior therapy
DBT: dialectical behavior therapy
MFQ: Mood and Feelings Questionnaire
NHS: National Health Service
NICE: National Institute of Health and Care Excellence
RCADS: Revised Child Anxiety and Depression Scale
SDQ: Strengths and Difficulties Questionnaire
